# Development of a Rapid Point-of-Use DNA Test for the Screening of Genuity® Roundup Ready 2 Yield® Soybean in Seed Samples

**DOI:** 10.1155/2016/3145921

**Published:** 2016-05-26

**Authors:** Dilip Chandu, Sudakshina Paul, Mathew Parker, Yelena Dudin, Jennifer King-Sitzes, Tim Perez, Don W. Mittanck, Manali Shah, Kevin C. Glenn, Olaf Piepenburg

**Affiliations:** ^1^Monsanto Company, 800 N. Lindbergh Boulevard, St. Louis, MO 63017, USA; ^2^TwistDx Limited, Minerva Building, Babraham Research Campus, Babraham, Cambridge CB22 3AT, UK

## Abstract

Testing for the presence of genetically modified material in seed samples is of critical importance for all stakeholders in the agricultural industry, including growers, seed manufacturers, and regulatory bodies. While rapid antibody-based testing for the transgenic protein has fulfilled this need in the past, the introduction of new variants of a given transgene demands new diagnostic regimen that allows distinguishing different traits at the nucleic acid level. Although such molecular tests can be performed by PCR in the laboratory, their requirement for expensive equipment and sophisticated operation have prevented its uptake in point-of-use applications. A recently developed isothermal DNA amplification technique, recombinase polymerase amplification (RPA), combines simple sample preparation and amplification work-flow procedures with the use of minimal detection equipment in real time. Here, we report the development of a highly sensitive and specific RPA-based detection system for Genuity Roundup Ready 2 Yield (RR2Y) material in soybean (*Glycine max*) seed samples and present the results of studies applying the method in both laboratory and field-type settings.

## 1. Introduction

The amplification and detection of signal from nucleic acid targets to test for the presence of specific genetic markers in sample material are of ever increasing importance in a vast array of application areas. These include trait detection applications in the agricultural sector, as well as clinical diagnostics, testing for food-borne pathogens, environmental testing, and many others [1–3].

Collectively, the field of nucleic acid based testing may be termed “molecular diagnostics,” and its central step most often consists of nucleic acid amplification techniques (NAATs). NAATs owe their increasing popularity to their extremely high sensitivity, specificity, speed, and operational simplicity. NAATs, in fact, increasingly complement or may replace traditional methods such as culturing techniques [4–6] and antibody-based protein detection techniques [7–9].

**Figure 1:**
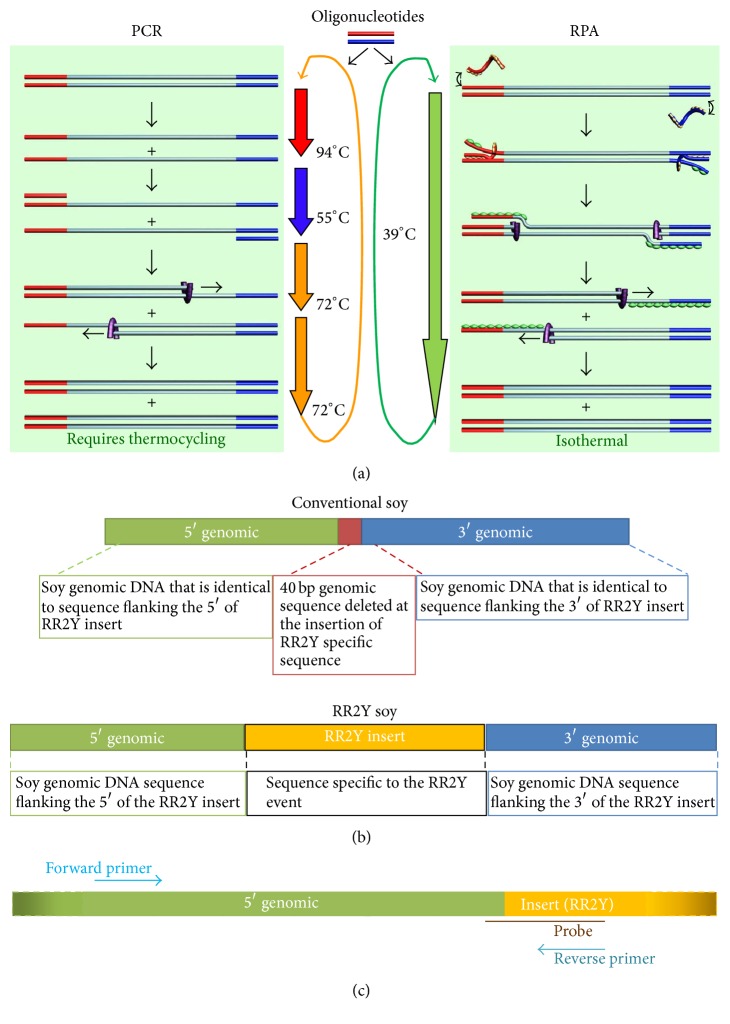
Target specific amplification of a DNA sequence using RPA. (a) Schematic of PCR and RPA processes. RPA uses recombinases and polymerase to bind and elongate the primers at a constant temperature resulting in the duplication of the target sequence. Multiple events of such duplication lead to exponential amplification under isothermal condition. On the other hand, PCR relies on thermal cycling and heat-stable polymerase for the amplification of the target sequence. (b) Organization of sequences at the site of RR2Y insertion in the conventional and RR2Y soybean genomes. (c) Arrangement of primers and probe used for RPA mediated amplification and detection of the RR2Y specific insertion.

Since its inception in the late 1980s, the polymerase chain reaction (PCR) has been the mainstay technique for the amplification of nucleic acids. Although PCR-based testing in laboratories is well established and extremely successful, the reliance of PCR on precise thermal control (at relatively high and rapidly changing—or “cycling”—temperatures; Figure 1(a)) has limited its use outside of centralized facilities. The thermocycling equipment used in PCR is expensive, particularly as suitable detection technologies have to be integrated with the amplification itself. Recent efforts to miniaturize and simplify PCR instrumentation have been reported [10–12]; however, these approaches have their limitations and have not been adopted in practice [13]. The limitations inherent in PCR make this method generally unsuitable for providing cost-effective access of molecular diagnostics to most end-users and have generally limited the adoption of NAAT-based diagnostic devices in point-of-use (POU)/point-of-care (POC) settings when dealing with noncentralized sample testing. Novel NAATs such as the isothermal recombinase polymerase amplification (RPA) [14], developed by TwistDx (Cambridge, UK), overcome the disadvantages of PCR-based technologies. RPA provides an inexpensive, fast, real-time, and reliable alternative POU/POC method that employs use of crude DNA preparation and simple field-deployable detection devices. Other technologies employing a constant temperature to facilitate the amplification of nucleic acids targets have also been reported in the literature. In contrast to PCR, the use of these isothermal technologies reduces the need for sophisticated instrumentation, consistent electrical power supply, and complex sample processing protocols [15, 16]. In addition to RPA, technologies utilizing amplification of nucleic acid targets at constant temperature such as nicking enzyme amplification reaction (NEAR) [17], loop mediated amplification (LAMP) [18], nucleic acid sequence based amplification (NASBA) [19], helicase dependent amplification (HDA) [20], and cross priming amplification (CPA) [21] have been reported in the literature. RPA is unique within this class of molecular methods in its combination of high performance (sensitivity and specificity of detection), low temperature operation, and overall robustness to temperature fluctuations [16, 22].

The expansion of molecular diagnostics from centralized laboratories into POU-type scenarios such as the one proposed in this publication requires the integration of NAAT platforms into low-cost and low-complexity devices with simple operating procedures. The isothermal NAAT RPA is ideally positioned to enable such solutions. RPA combines constant low reaction temperature with high sensitivity, specificity, and reaction speed. Briefly, in RPA, the sequence of biochemical events that facilitate the amplification of specific DNA fragments include binding of oligonucleotide primers to the target, extension of the bound primers by a DNA polymerase, and dissociation of the amplified product under isothermal condition (Figure 1(a)) [14].

As part of the RPA reaction, oligonucleotides used for amplification are mixed with DNA binding proteins (GP32, UvsX, and UvsY) resulting in formation of filaments of protein coated oligonucleotide complexes. Binding of GP32 to the oligos is followed by UvsY and finally UvsX, the recombinase. The UvsX/oligo complex searches the template DNA for homologous sequences and subsequently extends the primers. Target amplification is detected with a dual labeled oligonucleotide probe, containing a fluorophore (either TAMRA or FAM) and quencher separated by an abasic site (Table 1), of complimentary sequence. When the probe binds to the amplified target DNA, exonuclease III cleaves the abasic site, and the fluorophore and quencher are separated resulting in a fluorescence signal proportional to the amount of amplified target DNA.

RPA has been successfully employed in a number of reports for both human IVD [23–27] and veterinary applications [28, 29]. Importantly, substrate templates suitable for RPA can be obtained from crudely treated sample material. For this reason, the simplicity of the nucleic acid amplification can be mirrored by uncomplicated and simple front-end sample preparation procedures.

In order to constitute a fully integrated diagnostic system for the detection of genetic traits, the chosen sample processing procedure and the RPA biochemistry have to be augmented by a compatible read-out approach that is capable of monitoring a signal generated by the target amplification event and thus delivering the diagnostic information. Moreover, this detection method has to maintain the overall operational simplicity and portability of the complete POC system. An extremely useful tool to achieve these requirements is the use of a novel type of fluorogenic oligonucleotide probe specifically designed for RPA, in combination with appropriate fluorescence detection equipment. This approach is described in greater detail elsewhere [14, 16].

One potential application for the use of NAAT-based diagnostic devices is to develop field detection methods for genetically modified (GMO) crops. Since the first commercialization in the USA in 1996, millions of farmers demonstrate confidence in the benefits of GMO crops as evident by high adoption rates [30], especially in three large-acreage crops: maize (*Zea mays L.*), soybean (*Glycine max*), and cotton (*Gossypium* sp.). Currently, GMO crops are grown in ~30 countries worldwide over an accumulated area of ~1.5 billion hectares [30].

As part of the stewardship efforts supporting the commercialization of GM crops, international organizations such as BIO (Biotechnology Industry Organization) developed policy guidance documents (https://www.bio.org/sites/default/files/Product-Launch-Stewardship-11272012.pdf) [31] which mandate, among others, development of a reliable detection method or test for use by growers, processors, and buyers prior to product commercialization to enable crop identity verification for intended use.

Protein-based lateral-flow devices (LFD) are the current gold standard for the rapid, on-site test format for GMO sample screening [8, 9, 32]. LFD-based screening is used at different points in the grain trade, including farms, grain elevators, and ports to test for different GMO traits to support consumer preferences and trade requirements. However, ongoing improvements to GMO crops have resulted in the introduction of given transgenes, such as the gene for expressing the glyphosate-tolerant CP4 variant of 5-enolpyruvylshikimate-3-phosphate synthase (CP4 EPSPS), into multiple GMO crops, in which case protein-based detection methods cannot distinguish between these various products. In addition, many new GMO crops involve the combination by conventional breeding of two or more transgenic events (i.e., single-locus insertions) to offer combinations of insect and herbicide tolerance genes to combat a wider range of pests and weeds than covered by the single events [30, 33]. Additionally, other new GMO crops do not express a protein to achieve the intended trait (for e.g., Vistive® Gold Soybean). In all of these new GMO crops, protein-based detection methods, such as LFD, cannot differentiate the various traits for field detection.

Monsanto's Genuity Roundup Ready 2 Yield (RR2Y) and its predecessor Roundup Ready® soybean present a unique case study for the application of RPA technology to distinguish the two products at the molecular level. The unique protein expressed in RR1 and RR2Y soybean is the same CP4 EPSPS, preventing the use of protein-based LFD method to differentiate the two products.

The current publication reports the use of new NAAT-based technology platform to develop a field detection method, using RR2Y-specific assay as an example. This represents the development of a rapid and inexpensive field-deployable detection method that can effectively differentiate between samples from different GMO events, in this case RR1 and RR2Y soybean that express the same CP4 EPSPS protein. The report also demonstrates the ease and reliability of the method starting from a laboratory sample such as purified DNA template or whole seed sample encountered in a field.

## 2. Materials and Methods

### 2.1. Preparation of Seed Extracts for RPA Reactions

Typically, 100 seeds were collected and crushed in Oster Blender (Model 4655; 600 watt, 3 speeds) with ice crushing blade (#4961 USA). One scoop (~90 mg) of the seed powder was transferred to a tube containing 4 mL of lysis buffer (0.2 M NaOH). Each tube was shaken for approximately 5 seconds and incubated at room temperature (RT) for 1 minute to let the particulates settle down.

### 2.2. DNA Extraction for PCR and RPA

DNA was extracted from ground seed using the ZE Plant/Seed DNA miniprep*™* kit (Zymo Research) using a modified method of the manufacturer's instructions. Briefly, 1.3 mL of DNA Binding Buffer (with *β*-mercaptoethanol (BME)) was added to each 2 mL screwcap tube, followed by addition of ~90 mg of ground seed sample. Seed solution was mixed in a Disruptor Genie for 2 minutes and centrifuged at 10,000 ×g for 2 minutes. Supernatant (900 *µ*L) was transferred to a Zymo-Spin IIC Column in a collection tube and centrifuged at 10,000 ×g for 2 minutes. Zymo-Spin IIC Column was transferred to a new collection tube and 200 *µ*L of DNA Pre-Wash Buffer was added, followed by centrifugation at 10,000 ×g for 1 minute. Plant/Seed DNA Wash Buffer (500 *µ*L) was added to the Zymo-Spin IIC Column and centrifuged at 10,000 ×g for 1 minute. DNA was eluted from the Zymo-Spin IIC Column by incubating in presence of 30 *µ*L of elution buffer at RT for 2 minutes, followed by centrifugation at 10,000 ×g for 30 sec.

### 2.3. RPA Assay Design

Oligonucleotide primers and probes used to develop the RPA assays were purchased from Biosearch Technologies (Novato, CA). The RPA reactions were typically carried out at 39°C, unless otherwise noted, using a device that maintains constant temperature combined with fluorescence detection (Twista®, TwistDx, UK). Reaction contents were mixed prior to amplification and again at five minutes during incubation. The output of the reaction was monitored in real time using the Twista Studio Software (TwistDx, UK) with fluorescence measurements taken every 20 seconds for a total of 15 (or 20) minutes [22].

RPA assays with purified DNA as the template were performed as described below. Lyophilized RR2Y RPA Exo pellets were obtained from TwistDx. The RR2Y RPA Exo pellets comprised all essential components including the critical recombinase and polymerase proteins, such as 900 ng/*µ*L gp32, 120 ng/*µ*L uvsX, and 30 ng/*µ*L uvsY, 0.5 U of ExoIII, and 0.25 U of POL. Assay pellets were generated by lyophilizing reaction mixtures containing RR2Y primers at 420 nM,* lec* primers at 240 nM, RR2Y and* lec* specific probes at 120 nM each, 100 ng/*µ*L creatine kinase, 5.5% PEG 35 K, 6% Trehalose, 2.5 mM ATP, 50 mM phosphocreatine, and 240 *µ*M of each dNTP. To initiate an amplification reaction, the lyophilized pellets were rehydrated with 46.5 *µ*L of rehydration buffer (1.5% PEG 35 K, 100 mM potassium acetate, 35 mM Tris-acetate pH 8.3, and 14 mM magnesium acetate). Tubes were spun down after thorough mixing by vortex. One *µ*L of purified DNA or a loop (~1 *µ*L) of seed extract or buffer alone for no template control (NTC) was added to the rehydrated pellets. Pellets were mixed by vortex and spun down and 2.5 *µ*L magnesium acetate was added (final reaction volume of 50 *µ*L) to initiate the reaction. Samples were mixed by vortex and spun down prior to transferring the tubes to the Twista fluorescent reader.

### 2.4. RPA Assay Analysis and Cutoff Values: Positive/Negative Calls

Preassigned cutoff values are determined based on statistical analysis of a large set of data. These cutoff values are used to generate an algorithm that can translate the fluorescent amplification curves into positive/negative/invalid calls by the Twista fluorescent reader. The algorithm considers background fluorescence values, onset of amplification, and the strength of the signal between two defined time points (5–8 minutes) to determine the results of the assay. Using this algorithm, Twista can report the assay results without any manual intervention.

### 2.5. PCR

PCR amplification was carried out in a thermocycler (Eppendorf Mastercycler Personal) using the following primer pairs.

The oligonucleotide primers were purchased from Integrated DNA Technologies (San Diego, CA).

PCR amplification of RR1 and* lec* was carried out using the Qiagen Multiplex PCR Mastermix (2x) and 100 ng of genomic DNA in a final reaction volume of 40 *µ*L. PCR amplification for RR2Y was carried out using the HotStart-it*™* Taq Mastermix (2x) and 100 ng of genomic DNA in a final reaction volume of 50 *µ*L. RR1 and RR2Y reactions were performed using 200 nM forward and reverse primers, whereas the* lec* reactions were performed using 100 nM primers. The following cycling conditions were used for RR1 and* lec* reactions: 95°C for 10 minutes, 44 cycles of 94°C for 15 seconds, 62°C for 30 seconds, 72°C for 30 seconds, and a final extension at 72°C for 5 minutes. The following cycling conditions were used for RR2Y reactions: 95°C for 2 minutes, 34 cycles of 94°C for 30 seconds, 60°C for 30 seconds, 72°C for 30 seconds, and a final extension at 72°C for 5 minutes.

The PCR products were mixed with 10x BlueJuice*™* Gel Loading Dye (Invitrogen) and run in 8-well 1% precast agarose gel containing 1% ethidium bromide. Pictures of agarose gels were captured using the Molecular Imager® Gel Doc*™* XR.

### 2.6. Lateral-Flow Strip Detection

For lateral-flow strip experiments, conventional, RR1, and RR2Y soybean were tested in triplicate with the QuickStix*™* Kit for Roundup Ready Soybean (Envirologix; Part AS 010-BGB) according to the manufacturer's protocol. Briefly, 100 soybean seeds were counted and ground on high speed for 20 seconds. Seed powder and water were mixed in a ratio of 1 : 5 (w/v) and manually shaken for 30 seconds. About 12 mL of the extract was transferred carefully in order not to collect any solids into a three-ounce sample cup. The bottom portion of the test strip was dipped in the extract for 5 minutes. Strips were photographed at the end of five minutes for documentation. A pink band corresponding to the Control band developed in all strips indicating that the strips have functioned properly. Presence of the desired product is indicated by the appearance of an additional pink band above the Control band.

### 2.7. Stability Protocol and Analysis Method

To determine long-term stability of RPA reaction pellets at different temperatures, all experimental parameters such as template, buffers, analyst, protocol, and detection devices were kept constant. Reaction pellets were stored at four different temperatures: ≤−15°C (reference), 2–8°C, 22–28°C (ambient), or 35–40°C. Reaction pellets were produced as a single batch, packaged in a dry-room facility as strips of 8 PCR tubes, vacuum-sealed, and shipped on dry ice. The ≤−15°C, 2–8°C, and 22–28°C (ambient) storage units were tracked via an automated system to ensure accurate temperatures. The 35–40°C storage unit was tracked on a weekly basis by manual recording. The reaction pellets from all four temperature conditions were tested at 18 defined intervals over a year.

At each time point, eight reactions were tested per storage condition including six reactions using 0.1% RR2Y genomic DNA as positive control template and two reactions using genomic DNA from conventional soybean. Positive, negative, or invalid calls were made based on a slope cutoff of 90 mV/min for FAM and 600 mV/min for TAMRA. Statistical analysis was used to determine overall results of stability experiments using the following analysis of variance (ANOVA) model:

(1) $$\begin{array}{c} y_{ijk}=\mu +\tau_{i}+\theta_{i\left(j\right)}+\delta_{k}+{\tau \delta }_{ik}+\varepsilon_{ijk}, \end{array}$$

where *y*_*ijk*_ is the observed response of the *j*th well of the *i*th temperature at the *k*th time; *μ* is the overall mean, *τ*_*i*_ is the effect of the *i*th temperature; *θ*_*i*(*j*)_ is the random effect of the *j*th well in the *i*th temperature; *δ*_*k*_ is the effect of the *k*th time; *τδ*_*ik*_ is the effect of the interaction between the *i*th temperature and the *k*th time; and *ε*_*ijk*_ is the residual error.

Pairwise comparisons of each time point to time zero were defined within the ANOVA model and tested using Dunnett's test at the 5% level. There were multiple cases where statistical significance did not indicate a lack of stability. For example, a significantly lower response at time *n* would be followed by a nonsignificant difference at time *n* + 1. As a result a biologically meaningful difference was also used to evaluate the data. When the average response reached half of the mean at time zero, the pellets were considered unstable.

## 3. Results and Discussion

This report describes an assay designed to detect a DNA sequence of a genetic element in the soybean genome coding for CP4 EPSPS that confers tolerance to the herbicide Roundup®.

The assay described here is designed in a duplex format to allow simultaneous amplification and detection of the RR2Y insertion and the endogenous soybean gene, lectin (*lec*), as an internal control. Amplification of an endogenous gene such as* lec* confirms (1) the general activity of the RPA amplification/detection “machinery” of the reaction; (2) the effectiveness of the sample preparation procedure; and (3) the input of the correct sample type (soybean in this case).

The DNA junction between the soybean genome and the inserted CP4 EPSPS cassette is well characterized, allowing RPA oligonucleotide primers and probes to be designed to detect the “junction sequences” unique to RR2Y.  Figure 1(b) depicts the differences in the sequences of the conventional soybean and RR2Y soybean genomes at the site of insertion. The junction sequences are unique to the soybean RR2Y event and therefore deliver a high degree of molecular specificity. A number of primers and probes were designed across the junction sequence and screened for the amplification of RR2Y target by measuring the fluorescence output of the appropriate detection probes. Similarly, a number of primers and probes were designed and screened against the* lec* gene sequence. After ranking the primer/probe combinations according to performance, one probe and one primer pair each were chosen for the amplification and detection of the RR2Y and* lec* targets by RPA. Sequences of the primers and probes used for the RR2Y duplex RPA assay are included in Table 1 (see Figure 1(c) for the localization of RR2Y primers and probe at the locus).

**Figure 2:**
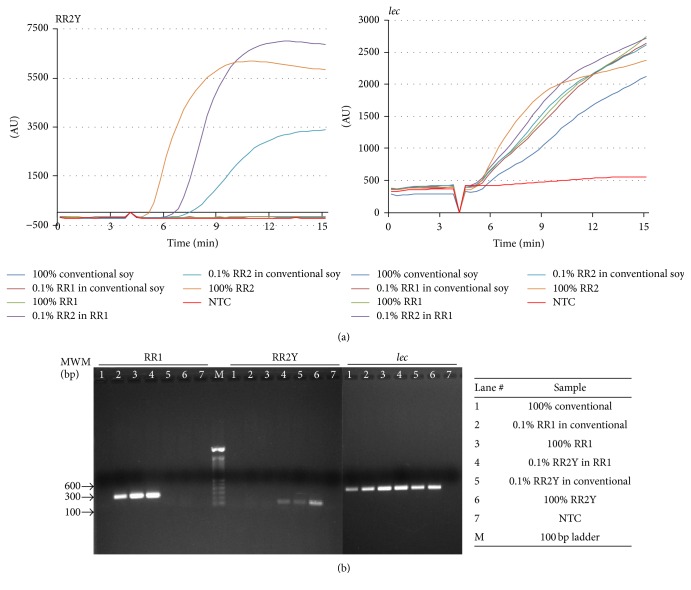
RPA mediated target specific amplification of RR2Y soybean DNA sequences. (a) Specificity of RPA mediated amplification of the endogenous* lec* or RR2Y specific insert sequences from different combinations of conventional, RR1, and RR2Y soybean DNA templates. (b) PCR followed by agarose gel electrophoresis of amplification products using* lec*, RR1, or RR2Y specific primers.

RPA reactions for RR2Y and* lec* targets were carried out and amplification of the targets was monitored fluorimetrically in real time using Twista portable fluorometer (TwistDx Limited, Cambridge, UK). Purified genomic DNA isolated from conventional, RR1, or RR2Y soybean seeds were used as templates to determine the specificity of the assay, as observed by detectable signal in 5–15 minutes (Figure 2(a)). The results demonstrate lack of amplification of the RR2Y target when conventional soybean DNA lacking the specific insertion was used as the amplification template. The transgenic inserts in RR1 and RR2Y have different junction sequences with the flanking soybean genome; therefore, amplification was not observed when RR1 DNA was used instead of RR2Y DNA as template. Exponential amplification was readily observed when RR2Y DNA was used as the template by itself (100% (w/w) RR2Y) or mixed with either the RR1 (0.1% (w/w) RR2Y in RR1) or the conventional DNA (0.1% (w/w) RR2Y in conventional). An earlier onset of amplification was observed when 100% (w/w) RR2Y DNA template was used instead of 0.1% (w/w) RR2Y, demonstrating that the onset of target detection is dependent upon the amount of target DNA. Amplification of endogenous* lec* target is observed when conventional soybean or RR1 or RR2Y soybean genomic DNA was used as the template indicating the presence of active reaction mix and sufficient template for amplification.

PCR reactions were performed with the same purified genomic DNA as above to confirm the identity of the samples. The PCR products were separated in an ethidium bromide stained agarose gel and visualized under a UV transilluminator (Figure 2(b) and Table 2). The PCR amplification products obtained using the RR2Y specific primers were consistent with the results of the RPA reactions. PCR was also performed using RR1-specific primers and specific amplification was observed using the RR1 soybean DNA as template. All samples, except for the reactions lacking any genomic DNA (no template control, NTC), displayed positive PCR amplification products with* lec* primers at the expected size range. These results combined with the RPA assay data confirm that the designed RPA assay is specific for DNA isolated from RR2Y soybean.

The specified level of discrimination of the RR2Y assay is to detect 0.5% (w/w) of RR2Y material in a mixture with 99.5% of non-RR2Y (conventional or RR1) soybean seed material. Due to the nature of the sample preparation procedure, in practice about 10–30 ng of total soybean DNA was analyzed per RPA reaction which corresponds to about 4000–12000 copies of genomic DNA. In a typical reaction at the specified discrimination level of 0.5% (w/w), 20–60 copies of the RR2Y target may be present to act as template for RPA. The copy numbers were calculated based on the genome size of soybean. In order to meet this requirement, first, the sensitivity of the RR2Y analyte detection must be high enough to be able to detect as low as 20 copies per reaction and, second, the assay must perform without significant loss of sensitivity, even in the presence of a comparatively large amount of total soybean DNA.

**Figure 3:**
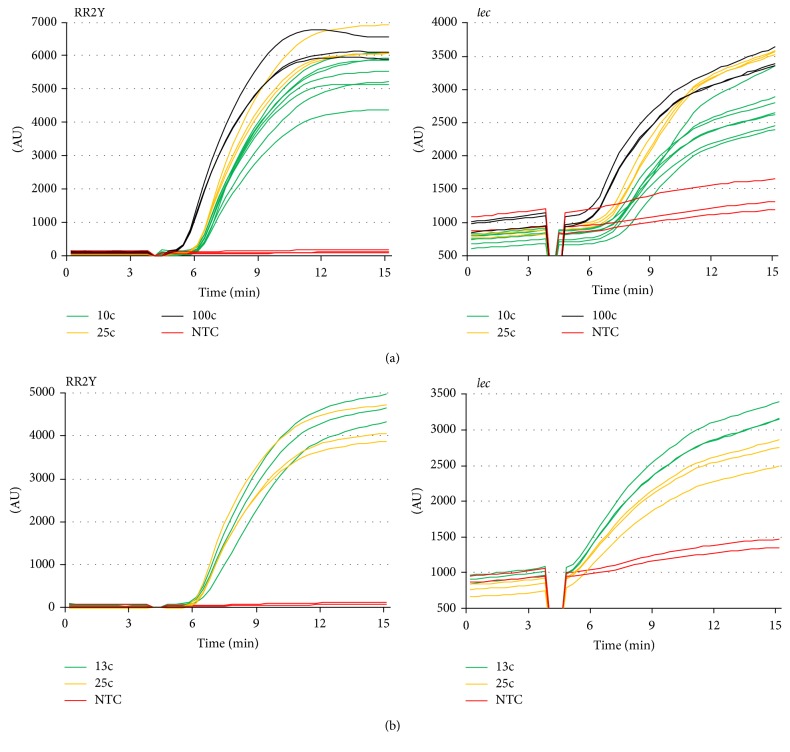
Sensitivity of RPA mediated target amplification. Duplex assays were performed with primers specific for endogenous* lec* (right panel) and RR2Y target (left panel) in the same reaction. Reactions without template DNA (NTC) were also carried out as a control. (a) Amplification curves of RPA reactions using DNA template at 100, 25, or 10 copies per reaction. (b) Amplification curves of RPA reactions using 25 or 13 copies of RR2Y DNA template per reaction in the presence of large amounts of conventional soybean DNA.

The sensitivity of the duplex RPA formulation was assessed by challenging it with different amounts of target DNA purified from RR2Y soybean seed samples as template (between 100 and 10 copies per reaction; see Figure 3(a)). A positive amplification signal was generated in all cases for the RR2Y containing samples, demonstrating that the sensitivity of the RR2Y is very high (e.g., positive detection was achieved in all 7 samples which contained 10 copies of RR2Y target). The time of onset of detection was approximately 5–7 minutes after the initiation of the reaction. Positive signals were also generated in all cases for the* lec* control reaction (Figure 3(a), right panel).

### 3.1. Sensitivity of the Duplex RPA Assay

The two individual RPA reactions of the combined duplex assay (RR2Y and* lec*) are to some degree in competition with each other (for nucleotides, recombinase protein, etc.). Additionally, at the discrimination level of 0.5%, a large imbalance of starting amount of template exists between the two component reactions, with the* lec* target being up to 200 times more numerous than the RR2Y analyte. These two factors need to be accounted for in the relative performance of the two assays. A duplex assay challenged with a low number of RR2Y target copies in the background of conventional soybean DNA would reflect the performance of the assay in a POU setting, compared with that described above (Figure 3(a)) where low copy numbers of purified RR2Y DNA have been used as template. A set of experiments were performed to determine the limit of RR2Y detection when challenging the assay with more complex DNA template. A 0.5% mixture of RR2Y in conventional soybean seeds (Figure 3(b)) was analyzed. Strong signals were still observed at RR2Y template amounts of 25 and 13 copies. Likewise, strong signals were also obtained for the* lec* control in all cases. The results demonstrate that the combined assay can detect the RR2Y target with sufficient sensitivity even if presented in the background of large amounts of nontarget DNA and despite the active amplification of the internal control,* lec*.

### 3.2. Robustness of RPA Reactions under Anticipated Field Conditions

Next, a series of experiments was conducted to analyze the robustness of the RPA assay using RR2Y soybean DNA template to determine its usability at the POU settings. Any assay that is to be deployed at such settings must be robust enough to provide consistent performance across varying conditions.

RPA reactions using the purified RR2Y DNA template at different temperatures were used to assess the effect of varying assay temperatures (37°C, 38°C, 40°C, 41°C, and the recommended 39°C) on the performance of the assay (Table 3). All the reactions were positive at the different temperatures tested, with an average onset time, range of 5.5–6.8 minutes, suggesting that the performance of the reaction is not affected by these variations.

**Figure 4:**
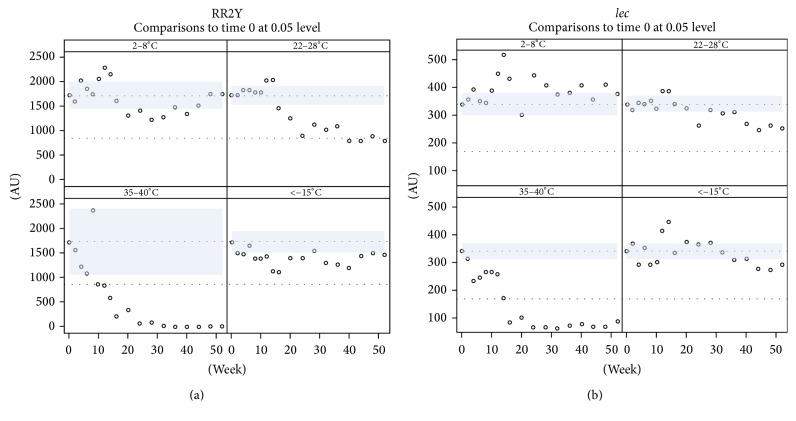
Robustness and stability of RPA reactions. (a, b) Long-term stability testing of the lyophilized RPA reaction pellets. Pellets were stored at different temperatures for up to one year and assays were conducted every three weeks. Dunnett test at 5% level was performed and mean fluorescence was plotted against time.

A time course experiment was performed to address the long-term storage stability of the RPA formulation. Many of the reagents that are critical for the RPA assay performance (such as proteins, oligonucleotides, and nucleotides) are potentially unstable, some of them even under refrigerated conditions, and are, therefore, provided in a lyophilized format as preformulated reaction pellets in microcentrifuge PCR strip tubes. Each strip of lyophilized RPA pellets was vacuum-sealed in a foil pouch. Sealed strips of lyophilized RPA reaction pellets were stored at different temperatures: in a freezer (<−15°C), in a refrigerator (2–8°C), at room temperature (RT; 22–28°C), and in an incubator (35–40°C). Eight reaction tubes from the respective storage temperature were tested for assay performance at regular intervals over a period of one year. RPA assays for the* lec* target were stable for one year in a freezer or refrigerator or at RT (Figure 4(b)). However, the performance of* lec* amplification/detection shown by pellets stored at elevated temperature in an incubator decreased after 20 weeks (Figure 4(b)). The RR2Y specific assay components were stable for the entire year when the pellets were stored in a refrigerator or a freezer. There was a pronounced drop in activity of the pellets with regard to the RR2Y assay, when the pellets were stored at RT. RR2Y assay performance, similar to the* lec* assay, dropped below useful levels, when the reaction pellets were stored at elevated temperatures in an incubator for 20 weeks or longer. In summary, RR2Y RPA reactions in the formulation tested are stable when stored in a freezer or refrigerator for up to one year and at RT for up to six months (Figure 4(a)). Increased stability characteristics may be achieved by introducing changes to the manufacturing process to increase dryness of the reaction pellets, or by changes to the reaction formulation, for example, by the inclusion of suitable additives. This is currently subject of further investigation and optimization.

### 3.3. RPA Assay in Field Conditions

**Figure 5:**
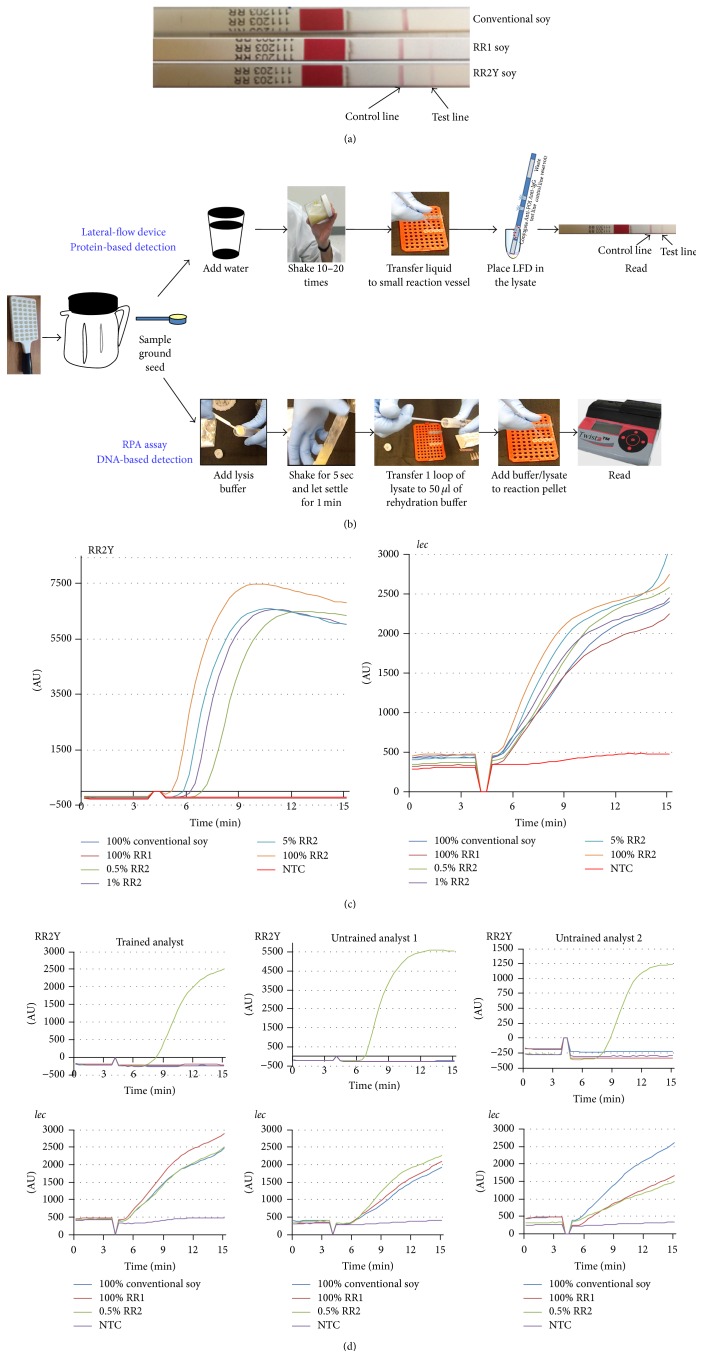
RPA-based field detection method. (a) Protein-based lateral-flow strips were used to detect conventional, RR1, and RR2Y yield soybean extracts. Genetic modification in the RR1 and RR2Y events produces the unique CP4 EPSPS protein not observed in the conventional soybean. (b) Flow chart depicting the sample processing and assay setup steps to perform the protein-based and RPA-based field detection methods. (c) Specificity and sensitivity of RPA-based RR2Y event specific amplification using crude seed extracts as the templates. Endogenous* lec* gene is also amplified in the duplex reaction as a control. (d) RR2Y soybean specific amplification reactions performed by trained (left) and untrained (middle and right) personnel to demonstrate the ease of carrying out RPA-based field detection assays.

To test the feasibility of RPA in POU setting, we performed a set of experiments. The current benchmark for field testing involves protein-based LFD that detects the presence of the target protein in crude seed extracts (Figure 5(a)). The unique DNA insertions in the RR1 and RR2Y genomes code for the same CP4 EPSPS protein which confers resistance to the herbicide Roundup. RR1, RR2Y, and conventional soybean seed extracts were tested using CP4 EPSPS specific protein strips. A faint pink line was observed with both RR1 and RR2Y seed samples in addition to the dark pink Control band seen with all the samples. Conventional seed samples showed development of Control band but not the test band, suggesting the test was performed appropriately and the conventional soybean sample is negative for the Roundup Ready trait (Figure 5(a)). In the POU setting, time and resources do not permit a laboratory-standard purification of genomic DNA from the seed samples. To utilize the RR2Y RPA assay as a field detection method, the reaction should be performed with simply derived seed extracts as the template material. Procedures were standardized to perform the RPA reactions starting with ground soybean seed as the source material (Figure 5(b)).

All samples tested showed positive amplification for the* lec* target (Figure 5(c), right panel). RR2Y primers and probe amplified the target from 100%, 5%, 1%, and 0.5% RR2Y seed samples (Figure 5(c), left panel). No amplification was observed with RR2Y primers and probe using conventional and RR1 soybean seed samples (Figure 5(c), left panel), demonstrating specificity and utility of the method for use in a field-setting. Additionally, the ease of use of the RR2Y RPA field detection method was tested. One trained (Figure 5(d), left panels) and two untrained analysts (Figure 5(d), middle and right panels) performed the reaction based on simple protocol provided to the analysts. All three analysts successfully performed the assay. The amplification of the internal control marker* lec* was observed with all soybean samples, except for the NTC (Figure 5(d), bottom panel). RR2Y specific amplification was observed with 0.5% (w/w) RR2Y samples (Figure 5(d), right panel) and, as expected, no amplification was observed with conventional, RR1 soybean seed extracts, or NTC (Figure 5(d), top panels).

In a POU setting, end-users will not need to analyze the reaction curves to determine the result of a test. Instruments and the associated software used to perform the current assay were developed so that the results of each of the reactions could also be analyzed by an algorithm preprogrammed and displayed on the screen as + (positive), − (negative), or ? (invalid) at the end of the reaction (data not shown). Results from the screen display matched the amplification curves (data not shown), as expected.

## 4. Conclusion

In summary, the present studies demonstrate that RPA-based field detection assays can be fast and simple and performed by a relatively untrained user. The ability of RPA assays to detect DNA sequences unique to GMO crops provides an advantage over LFD for GMO crops that share the same protein. RPA assays also have intrinsic advantages over PCR assays. RPA assays are simpler, easier, and faster than PCR assays for an end-user. Instrumentation used for the RPA-based assays is relatively cheaper than a PCR thermocycler, largely owing to the isothermal nature of the RPA reaction. In particular, the current report presents an RPA-based field detection method that can specifically detect RR2Y soybean. The assay was developed in a duplex format allowing simultaneous detection of the endogenous* lec* gene as a control with soybean samples, irrespective of the specific trait(s) of interest for a given RPA assay.

Detection of a number of novel traits could benefit from employing RPA, including RNA interference-based traits where the inserted DNA in the transgenic event does not encode a protein (Vistive® Gold Soybean). RPA technology provides benefit to products with multiple stacked traits by delivering insertion-specific detection of the individual traits. The technology offers a significant breakthrough for the development of detection methods at the elevators and ports and in the field to ensure regulatory and product stewardship compliance and validate product claims.

## Figures and Tables

**Table 1:** Sequences of RPA and PCR primers and probes used in the study.

Assay	Description	Sequence
RPA	RR2Y probe	cccgccttcagtttaaactatcagtgtttggagc-T(BHQ^a^-2)-t-dSpacer-a-T(TAMRA^b^)-aaccacgattgaag
	RR2Y forward primer	ccctcttggcttttctaagtttgagctcgttactg
	RR2Y reverse primer	cccgccttcagtttaaactatcagtgtttgg
	*lec* probe	ggaaactgtttctttcagctggaacaag-T(FAM^c^)-t-dSpacer-g-T(BHQ-1)-gccgaagcaacc
	*lec* forward primer	ccagaatgtggttgtatctctctccctaacctt
	*lec* reverse primer	cccgaggaggtcacaatagcgtctccttggag

PCR	RR1 forward primer	tttgggaccactgtcggcagaggcatctt
	RR1 reverse primer	gatttgaattcagaaccttgtgca
	RR2Y forward primer	tcccgctctagcgcttcaat
	RR2Y reverse primer	tcgagcaggacctgcagaa
	*lec* forward primer	gtttgacactttccggaactcttg
	*lec* reverse primer	ctgtcacatttagatggcctcatg

^a^BHQ = dT Black Hole Quencher; ^b^TAMRA = dT TAMRA; ^c^FAM = dT FAM.

**Table 2:** Table summarizing the expected sizes of the amplification products and experimental results of PCR and RPA mediated amplification reactions.

Assays	Expected size (bp)	Conv	RR1 in Conv	RR1	RR2Y in RR1	RR2Y in Conv	RR2Y	NTC
RR1 PCR	275	−	+	+	+	−	−	−
RR2Y PCR	139	−	−	−	+	+	+	−
*lec* PCR	400	+	+	+	+	+	+	−
RR2Y RPA	N/A	−	−	−	+	+	+	−

**Table 3:** RPA assays were performed at the optimized target temperature of 39°C or at temperatures ±2°C of the target. Robust amplification was observed at all the temperatures tested.

Temperature (°C)	Onset time (min; *n* = 6)
37	6.3–7.3
38	5.7–6.7
39	5.7–6.3
40	5.3–6.0
41	5.3–5.7
